# A semi-analytical passive strategy to examine the water-ethylene glycol (50:50)-based hybrid nanofluid flow over a spinning disk with homogeneous–heterogeneous reactions

**DOI:** 10.1038/s41598-022-21080-z

**Published:** 2022-10-12

**Authors:** Ebrahem A. Algehyne, Nifeen H. Altaweel, Anwar Saeed, Abdullah Dawar, Muhammad Ramzan, Poom Kumam

**Affiliations:** 1 Department of Mathematics, Faculty of Science, University of Tabuk, P.O. Box 741, Tabuk, 71491 Saudi Arabia; 2 Nanotechnology Research Unit (NRU), University of Tabuk, Tabuk, 71491 Saudi Arabia; 3 Center of Excellence in Theoretical and Computational Science (TaCS-CoE), Science Laboratory Building, Faculty of Science, King Mongkut’s University of Technology Thonburi (KMUTT), 126 Pracha-Uthit Road, Bang Mod, Thung Khru, Bangkok, 10140 Thailand; 4 Department of Mathematics, Abdul Wali Khan University, Mardan, 23200 Khyber Pakhtunkhwa Pakistan; 5 Department of Medical Research, China Medical University Hospital, China Medical University, Taichung, 40402 Taiwan

**Keywords:** Engineering, Mathematics and computing

## Abstract

Scientists and researchers are much interested in studying graphene and silver nanoparticles for the enhancement of heat transport due to their extensive variety of applications in different areas of industrial and engineering such as drug delivery, medical devices, ultra-light, excellent electrical conductivity, strong medical strength, health care, consumer, food, etc. Therefore, in the existing investigation, the MHD flow of a mixed convective hybrid nanoliquid with graphene and silver nanoparticles past a rotating disk is considered. Water and ethylene glycol (50:50) is used as a base liquid in the existing model. The mechanism for heat transport is computed with the existence of thermal radiation and thermal convective condition. Homogeneous and heterogeneous chemical reactions are assumed in the flow behavior. The mathematical formulation of the proposed problem is based on the nonlinear PDEs which are then transformed to nonlinear ODEs by manipulating the appropriate similarity transformation. The simulation of the existing problem has been performed with the help of the homotopy analysis technique. The outcomes of the different flow parameters on the velocities, temperature, concentration, skin friction coefficient, and Nusselt number of the hybrid nanofluid are attained via graphs and tables. Some significant results from the existing problem demonstrate that the rate of heat transport is greater for the thermal Biot number and nanoparticles volume fraction. Further, it is noticed that the velocity of the liquid particles becomes lower for a higher magnetic field parameter.

## Introduction

In these days numerous processes at industrial level encompass of thermal energy transfer. In any industrial or engineering phenomena heat is required to be removed, added or transferred from one stream to another one, which has become a key task in industry. There are many methods used to enhance thermal performance, one of which is the spread of nano-sized particles in pure fluid as proposed by Choi and Estman^1^. Shah et al.^2^ have deliberated upon micropolar fluid flow with gold nanoparticles in the blood and have established that fluid micro-rotational velocity has amplified with growing in rotational factor while thermal flow of blood has grown up with enhancement in nanoparticles concentration. Shoaib et al.^3^ computed intelligently the generation of irreversibility and thermal flow rate for radiated nanoliquid flow over a sheet. Yadav et al.^4^ have inspected the impacts of magnetic field and sinusoidal heating on nanoparticles flow through a cavity. Khan et al.^5^ have deliberated radiative swirling motion of MHD Casson nanofluid over gyrating cylinder. Safwa et al.^6^ have used non-Fourier energy modal to discuss nanofluid flow over shrinking/stretching porous surface and have evaluated dual solution of their modeled problem. Biswas et al.^7^ have evaluated computationally the influence of MHD over Maxwell nanoparticles flow past a stretching sheet subject to thermally radiated chemical reactions.

The mixing of two different nanoparticles in pure fluid further supplements the thermal performance of pure fluid. Wahid et al.^8^ have studied Marangoni hybrid nanoparticles flow on an infinite penetrable disk and have revealed that thermal flow has accelerated with augmentation in nanoparticles volume fraction, suction and porosity factors. As depicted by Usman et al.^9^ the hybrid nanoparticles have superior thermal flow rate in comparison of nanofluid. Sundaeep et al.^10^ have deliberated influence of nonlinear thermal radiations upon MHD hybrid nanofluid flow subject to effects of heat source on a curved stretching sheet. Ashwinkumar et al.^11^ have deliberated convective thermal flow in MHD hybrid nanoparticles using two different geometrical views and have concluded that augmentation in thermal radiation factor and nanoparticles concentration have boosted the distributions of temperature. Chu et al.^12^ have scrutinized MHD time-dependent hybrid nanofluid flowing amid of infinite two plates subject to different shape of nanoparticles. Khan et al.^13^ discussed bioconvection hybrid nanoliquid flow upon a heated thin movable needle. Waini et al.^14^ have studied MHD time-dependent hybrid nanoparticles flow subject to the motion of fluid induced by rotary disk. Eid and Nafe^15^ have analyzed variations in thermal conductance and effects of thermal flow generation over MHD hybrid nanofluid flow on permeable surface using slip conditions.

Mixed convective flows of fluid also called combined natural and forced convective flows occur in many transportation phenomena at industrial level. These flows are very important and play a pivotal role in many industrial applications. Alghamdi^16^ has inspected the influences of chemically reactive activation energy upon mixed convective nanoparticles flow past a rotary disk and have concluded that thermal and mass diffusivities have upsurge with augmentation in Hartmann number. Ahmad et al.^17^ have evaluated mixed convection liquid flow above a curved surface subject to influences of chemical reactions. Qaiser et al.^18^ assisted numerically the mixed convection fluid flow past an expanding surface and have revealed that motion of fluid has retarded and heat flow has upsurge with higher values of magnetic field effects. Rasool and Wakif^19^ have scrutinized numerically the effects of mixed convective EMHD over nanofluid flow over Riga plate by employing modified Buongiorno nanoparticles model. Awan et al.^20^ have produced a scientific result for rate type fluid using the effects of mixed convection upon fluid flow system through a singular kernel. Islam et al.^21^ have investigated mixed convective Maxwell nanofluid flow past an extending cylinder, using different flow conditions with their impact upon flow system.

The investigation of magnetic characteristics and electrically conducting behavior of fluid is termed as magneto-hydrodynamics (MHD) included salty water, plasma and liquid metals etc. Behind MHD the focal idea is that current can be persuaded by employing magnetic effects that causes polarization of liquids as proposed by Alfven^22^. Many applications of these fluids comprised the fields of astrophysics, geophysics magnetic drug delivery and sensor engineering etc. Sohail et al.^23^ have presented MHD Casson liquid flow subject to thermal conductance past a bidirectional nonlinear stretching surface. Khan et al.^24^ have explored MHD nanoliquid flow with influence of chemically reactive and viscously dissipative effects past a moving needle. Kodi and Mupori^25^ have deliberated time-dependent Casson MHD fluid flow past a surface using chemical reactions and heat absorption influences upon flow system. Nazeer et al.^26^ deliberated theoretically the MHD liquid flow in a micro-channel and have revealed that velocity of fluid flow system has retarded in response of strong magnetic field. Asjad et al.^27^ inspected the effects of activation energy upon bioconvective MHD liquid flow through an exponentially expanding sheet.

Heat transmission is mostly boosted by applying thermal radiations to the fluid flow system. These radiations play a substantial role in heat flow problems when coefficient of convective heat transfer is pretty small. Waqas et al.^28^ have analyzed the significances of chemically reactive and bio-convective on MHD nanofluid flow using thermal radiation and have determined that heat flow of fluid has inflamed with magnetic effects and radiation factor. Muhammad et al.^29^ have inspected nonlinear thermal radiated Eyring-Powel nanoparticles flow above a Riga plate. Kumar et al.^30^ have discussed the thermal radiation influence upon MHD naturally convective nanofluid flow past a plate using sudden jerk. Shaw et al.^31^ have discussed MHD hybrid nanoliquid flow subject to the effects of quadratic thermal radiation above a stretched cylinder. Gumber et al.^32^ have scrutinized micropolar nanoliquid flow over a vertically placed plate with consequence of thermal radiations and suction/injection effects. Wahid et al.^33^ have deliberated MHD mixed convection nanoliquid flow past an absorbent flat vertical plate using thermal radiations.

Many fluid flow systems with influence of chemical reactions are incorporating both homogenous as well heterogeneous characteristics for instance biochemical reactions combustions and catalysis etc. Wang et al.^34^ have inspected the influence of homogenous /heterogeneous reactions upon a non-Newtonian fluid flow. Bashir et al.^35^ have conducted a comparative work on nanofluid flow using homogeneous and heterogeneous influence and have concluded that with augmentation in nanoparticles concentration, the thermal profiles and skin friction have upsurge. Nawaz et al.^36^ have reviewed numerically the effects of Darcy-Forchheimer model on micropolar fluid with homogeneous and heterogeneous reactions. Gangadhar et al.^37^ have deliberated MHD fluid flow with influences of autocatalyze chemical reactions and convection heat effects. Tahir et al.^38^ have investigated numerically the influences of heterogeneous and homogenous reactions upon Maxwell nanofluid flow over stretched surface subject to magnetic effects. Ayub et al.^39^ have deliberated MHD nanoliquid flow through gyratory disks subject to heterogeneous and homogenous reactions and have established that liquid motion has retarded and temperature profiles have upsurge with corresponding incrimination in the values of magnetic parameter.

From the above cited literature, it is observed that there is less work on the enhancement of heat transport of the water and ethylene glycol (50:50)-based hybrid nanofluid flow containing silver and graphene nanoparticles over a rotating disk. Therefore, in the present work for the improvement of heat transport, the graphene and silver nanoparticles are mixed in the base fluid 50%W (water) + 50%EG (ethylene glycol) over a rotating disk is considered. The role of magnetic field and thermal radiation are taken into deliberation. The catalyzed chemical reactions including the homogeneous and heterogeneous chemical reactions are evaluated in the current analysis. With the implementation of HAM scheme, the solution to the present problem is obtained. Flow behavior of various profiles of the hybrid nanoliquid versus discrete flow parameters is accomplished via graphs. The skin friction and heat transmission of the hybrid nanofluid with respect to different parameters are also discussed.

## Problem formulation

Let us assume the mixed convective flow of a hybrid nanofluid over a spinning disk with magnetic field and thermal radiation effects. The flow is taken as laminar, steady, and incompressible. Simulation for mass transport is encountered with the utilization of homogeneous and heterogeneous chemical reactions. In the present study, graphene and silver nanoparticles are mixed with 50%W (Water) + 50%EG (Ethylene glycol). It is assumed that the disk rotates with the angular velocity $$\overset{\lower0.5em\hbox{$\smash{\scriptscriptstyle\frown}$}}{\Omega }$$ along $$\overset{\lower0.5em\hbox{$\smash{\scriptscriptstyle\frown}$}}{z}$$-axis. In a cylindrical coordinate system, the velocity components are denoted by $$\left( {\overset{\lower0.5em\hbox{$\smash{\scriptscriptstyle\frown}$}}{r} ,\overset{\lower0.5em\hbox{$\smash{\scriptscriptstyle\frown}$}}{\vartheta } ,\overset{\lower0.5em\hbox{$\smash{\scriptscriptstyle\frown}$}}{z} } \right)$$. A magnetic field of strength $$B_{0}$$ is employed normal to the flow direction. The convective condition is implemented for the simulation of heat transport. The surface temperature is represented by $$\overset{\lower0.5em\hbox{$\smash{\scriptscriptstyle\frown}$}}{T}_{w}$$ and $$\overset{\lower0.5em\hbox{$\smash{\scriptscriptstyle\frown}$}}{T}_{\infty }$$ represents the ambient temperature. Keeping in mind the aforementioned flow assumptions, the governing equations can be written as^40^:

1 $$ \frac{{\partial \overset{\lower0.5em\hbox{$\smash{\scriptscriptstyle\frown}$}}{u} }}{{\partial \overset{\lower0.5em\hbox{$\smash{\scriptscriptstyle\frown}$}}{r} }} + \frac{{\overset{\lower0.5em\hbox{$\smash{\scriptscriptstyle\frown}$}}{u} }}{{\overset{\lower0.5em\hbox{$\smash{\scriptscriptstyle\frown}$}}{r} }} + \frac{{\partial \overset{\lower0.5em\hbox{$\smash{\scriptscriptstyle\frown}$}}{w} }}{{\partial \overset{\lower0.5em\hbox{$\smash{\scriptscriptstyle\frown}$}}{z} }} = 0, $$

2 $$ \overset{\lower0.5em\hbox{$\smash{\scriptscriptstyle\frown}$}}{\rho }_{hnf} \left( {\overset{\lower0.5em\hbox{$\smash{\scriptscriptstyle\frown}$}}{u} \frac{{\partial \overset{\lower0.5em\hbox{$\smash{\scriptscriptstyle\frown}$}}{u} }}{{\partial \overset{\lower0.5em\hbox{$\smash{\scriptscriptstyle\frown}$}}{r} }} - \frac{{\overset{\lower0.5em\hbox{$\smash{\scriptscriptstyle\frown}$}}{v}^{2} }}{{\overset{\lower0.5em\hbox{$\smash{\scriptscriptstyle\frown}$}}{r} }} + \overset{\lower0.5em\hbox{$\smash{\scriptscriptstyle\frown}$}}{w} \frac{{\partial \overset{\lower0.5em\hbox{$\smash{\scriptscriptstyle\frown}$}}{u} }}{{\partial \overset{\lower0.5em\hbox{$\smash{\scriptscriptstyle\frown}$}}{z} }}} \right) = \overset{\lower0.5em\hbox{$\smash{\scriptscriptstyle\frown}$}}{\mu }_{hnf} \left( {\frac{{\partial^{2} \overset{\lower0.5em\hbox{$\smash{\scriptscriptstyle\frown}$}}{u} }}{{\partial \overset{\lower0.5em\hbox{$\smash{\scriptscriptstyle\frown}$}}{r}^{2} }} + \frac{1}{{\overset{\lower0.5em\hbox{$\smash{\scriptscriptstyle\frown}$}}{r} }}\frac{{\partial \overset{\lower0.5em\hbox{$\smash{\scriptscriptstyle\frown}$}}{u} }}{{\partial \overset{\lower0.5em\hbox{$\smash{\scriptscriptstyle\frown}$}}{r} }} - \frac{{\overset{\lower0.5em\hbox{$\smash{\scriptscriptstyle\frown}$}}{u} }}{{\overset{\lower0.5em\hbox{$\smash{\scriptscriptstyle\frown}$}}{r}^{2} }} + \frac{{\partial^{2} \overset{\lower0.5em\hbox{$\smash{\scriptscriptstyle\frown}$}}{u} }}{{\partial \overset{\lower0.5em\hbox{$\smash{\scriptscriptstyle\frown}$}}{z}^{2} }}} \right) - \overset{\lower0.5em\hbox{$\smash{\scriptscriptstyle\frown}$}}{\sigma }_{hnf} B_{0}^{2} \overset{\lower0.5em\hbox{$\smash{\scriptscriptstyle\frown}$}}{u} + g\left( {\overset{\lower0.5em\hbox{$\smash{\scriptscriptstyle\frown}$}}{\rho } \overset{\lower0.5em\hbox{$\smash{\scriptscriptstyle\frown}$}}{\beta } } \right)_{hnf} \left( {\overset{\lower0.5em\hbox{$\smash{\scriptscriptstyle\frown}$}}{T} - \overset{\lower0.5em\hbox{$\smash{\scriptscriptstyle\frown}$}}{T}_{\infty } } \right), $$

3 $$ \overset{\lower0.5em\hbox{$\smash{\scriptscriptstyle\frown}$}}{\rho }_{hnf} \left( {\overset{\lower0.5em\hbox{$\smash{\scriptscriptstyle\frown}$}}{u} \frac{{\partial \overset{\lower0.5em\hbox{$\smash{\scriptscriptstyle\frown}$}}{v} }}{{\partial \overset{\lower0.5em\hbox{$\smash{\scriptscriptstyle\frown}$}}{r} }} + \frac{{\overset{\lower0.5em\hbox{$\smash{\scriptscriptstyle\frown}$}}{u} \overset{\lower0.5em\hbox{$\smash{\scriptscriptstyle\frown}$}}{v} }}{{\overset{\lower0.5em\hbox{$\smash{\scriptscriptstyle\frown}$}}{r} }} + \overset{\lower0.5em\hbox{$\smash{\scriptscriptstyle\frown}$}}{w} \frac{{\partial \overset{\lower0.5em\hbox{$\smash{\scriptscriptstyle\frown}$}}{v} }}{{\partial \overset{\lower0.5em\hbox{$\smash{\scriptscriptstyle\frown}$}}{z} }}} \right) = \overset{\lower0.5em\hbox{$\smash{\scriptscriptstyle\frown}$}}{\mu }_{hnf} \left( {\frac{{\partial^{2} \overset{\lower0.5em\hbox{$\smash{\scriptscriptstyle\frown}$}}{v} }}{{\partial \overset{\lower0.5em\hbox{$\smash{\scriptscriptstyle\frown}$}}{r}^{2} }} + \frac{1}{{\overset{\lower0.5em\hbox{$\smash{\scriptscriptstyle\frown}$}}{r} }}\frac{{\partial \overset{\lower0.5em\hbox{$\smash{\scriptscriptstyle\frown}$}}{v} }}{{\partial \overset{\lower0.5em\hbox{$\smash{\scriptscriptstyle\frown}$}}{r} }} - \frac{{\overset{\lower0.5em\hbox{$\smash{\scriptscriptstyle\frown}$}}{v} }}{{\overset{\lower0.5em\hbox{$\smash{\scriptscriptstyle\frown}$}}{r}^{2} }} + \frac{{\partial^{2} \overset{\lower0.5em\hbox{$\smash{\scriptscriptstyle\frown}$}}{v} }}{{\partial \overset{\lower0.5em\hbox{$\smash{\scriptscriptstyle\frown}$}}{z}^{2} }}} \right) - \overset{\lower0.5em\hbox{$\smash{\scriptscriptstyle\frown}$}}{\sigma }_{hnf} B_{0}^{2} \overset{\lower0.5em\hbox{$\smash{\scriptscriptstyle\frown}$}}{v} , $$

4 $$ \left( {\overset{\lower0.5em\hbox{$\smash{\scriptscriptstyle\frown}$}}{\rho } \overset{\lower0.5em\hbox{$\smash{\scriptscriptstyle\frown}$}}{C}_{{\overset{\lower0.5em\hbox{$\smash{\scriptscriptstyle\frown}$}}{p} }} } \right)_{hnf} \left( {\overset{\lower0.5em\hbox{$\smash{\scriptscriptstyle\frown}$}}{u} \frac{{\partial \overset{\lower0.5em\hbox{$\smash{\scriptscriptstyle\frown}$}}{T} }}{{\partial \overset{\lower0.5em\hbox{$\smash{\scriptscriptstyle\frown}$}}{r} }} + \overset{\lower0.5em\hbox{$\smash{\scriptscriptstyle\frown}$}}{w} \frac{{\partial \overset{\lower0.5em\hbox{$\smash{\scriptscriptstyle\frown}$}}{T} }}{{\partial \overset{\lower0.5em\hbox{$\smash{\scriptscriptstyle\frown}$}}{z} }}} \right) = \overset{\lower0.5em\hbox{$\smash{\scriptscriptstyle\frown}$}}{k}_{hnf} \left( {\frac{{\partial^{2} \overset{\lower0.5em\hbox{$\smash{\scriptscriptstyle\frown}$}}{T} }}{{\partial \overset{\lower0.5em\hbox{$\smash{\scriptscriptstyle\frown}$}}{r}^{2} }} + \frac{1}{{\overset{\lower0.5em\hbox{$\smash{\scriptscriptstyle\frown}$}}{r} }}\frac{{\partial \overset{\lower0.5em\hbox{$\smash{\scriptscriptstyle\frown}$}}{T} }}{{\partial \overset{\lower0.5em\hbox{$\smash{\scriptscriptstyle\frown}$}}{r} }} + \frac{{\partial^{2} \overset{\lower0.5em\hbox{$\smash{\scriptscriptstyle\frown}$}}{T} }}{{\partial \overset{\lower0.5em\hbox{$\smash{\scriptscriptstyle\frown}$}}{z}^{2} }}} \right) - \frac{{\partial \overset{\lower0.5em\hbox{$\smash{\scriptscriptstyle\frown}$}}{q}_{r} }}{{\partial \overset{\lower0.5em\hbox{$\smash{\scriptscriptstyle\frown}$}}{z} }}, $$

5 $$ \overset{\lower0.5em\hbox{$\smash{\scriptscriptstyle\frown}$}}{u} \frac{{\partial \overset{\lower0.5em\hbox{$\smash{\scriptscriptstyle\frown}$}}{A} }}{{\partial \overset{\lower0.5em\hbox{$\smash{\scriptscriptstyle\frown}$}}{r} }} + \overset{\lower0.5em\hbox{$\smash{\scriptscriptstyle\frown}$}}{w} \frac{{\partial \overset{\lower0.5em\hbox{$\smash{\scriptscriptstyle\frown}$}}{A} }}{{\partial \overset{\lower0.5em\hbox{$\smash{\scriptscriptstyle\frown}$}}{z} }} = \overset{\lower0.5em\hbox{$\smash{\scriptscriptstyle\frown}$}}{D}_{{\overset{\lower0.5em\hbox{$\smash{\scriptscriptstyle\frown}$}}{A} }} \left( {\frac{{\partial^{2} \overset{\lower0.5em\hbox{$\smash{\scriptscriptstyle\frown}$}}{A} }}{{\partial \overset{\lower0.5em\hbox{$\smash{\scriptscriptstyle\frown}$}}{r}^{2} }} + \frac{1}{{\overset{\lower0.5em\hbox{$\smash{\scriptscriptstyle\frown}$}}{r} }}\frac{{\partial \overset{\lower0.5em\hbox{$\smash{\scriptscriptstyle\frown}$}}{A} }}{{\partial \overset{\lower0.5em\hbox{$\smash{\scriptscriptstyle\frown}$}}{r} }} + \frac{{\partial^{2} \overset{\lower0.5em\hbox{$\smash{\scriptscriptstyle\frown}$}}{A} }}{{\partial \overset{\lower0.5em\hbox{$\smash{\scriptscriptstyle\frown}$}}{z}^{2} }}} \right) - \overset{\lower0.5em\hbox{$\smash{\scriptscriptstyle\frown}$}}{K}_{C} \overset{\lower0.5em\hbox{$\smash{\scriptscriptstyle\frown}$}}{A} \overset{\lower0.5em\hbox{$\smash{\scriptscriptstyle\frown}$}}{B}^{2} , $$

6 $$ \overset{\lower0.5em\hbox{$\smash{\scriptscriptstyle\frown}$}}{u} \frac{{\partial \overset{\lower0.5em\hbox{$\smash{\scriptscriptstyle\frown}$}}{B} }}{{\partial \overset{\lower0.5em\hbox{$\smash{\scriptscriptstyle\frown}$}}{r} }} + \overset{\lower0.5em\hbox{$\smash{\scriptscriptstyle\frown}$}}{w} \frac{{\partial \overset{\lower0.5em\hbox{$\smash{\scriptscriptstyle\frown}$}}{B} }}{{\partial \overset{\lower0.5em\hbox{$\smash{\scriptscriptstyle\frown}$}}{z} }} = \overset{\lower0.5em\hbox{$\smash{\scriptscriptstyle\frown}$}}{D}_{{\overset{\lower0.5em\hbox{$\smash{\scriptscriptstyle\frown}$}}{B} }} \left( {\frac{{\partial^{2} \overset{\lower0.5em\hbox{$\smash{\scriptscriptstyle\frown}$}}{B} }}{{\partial \overset{\lower0.5em\hbox{$\smash{\scriptscriptstyle\frown}$}}{r}^{2} }} + \frac{1}{{\overset{\lower0.5em\hbox{$\smash{\scriptscriptstyle\frown}$}}{r} }}\frac{{\partial \overset{\lower0.5em\hbox{$\smash{\scriptscriptstyle\frown}$}}{B} }}{{\partial \overset{\lower0.5em\hbox{$\smash{\scriptscriptstyle\frown}$}}{r} }} + \frac{{\partial^{2} \overset{\lower0.5em\hbox{$\smash{\scriptscriptstyle\frown}$}}{B} }}{{\partial \overset{\lower0.5em\hbox{$\smash{\scriptscriptstyle\frown}$}}{z}^{2} }}} \right) + \overset{\lower0.5em\hbox{$\smash{\scriptscriptstyle\frown}$}}{K}_{C} \overset{\lower0.5em\hbox{$\smash{\scriptscriptstyle\frown}$}}{A} \overset{\lower0.5em\hbox{$\smash{\scriptscriptstyle\frown}$}}{B}^{2} , $$

with boundary conditions^46,47^:

7 $$ \left\{ \begin{gathered} \overset{\lower0.5em\hbox{$\smash{\scriptscriptstyle\frown}$}}{u} = 0,\quad \overset{\lower0.5em\hbox{$\smash{\scriptscriptstyle\frown}$}}{v} = \overset{\lower0.5em\hbox{$\smash{\scriptscriptstyle\frown}$}}{\Omega } \overset{\lower0.5em\hbox{$\smash{\scriptscriptstyle\frown}$}}{r} ,\quad \overset{\lower0.5em\hbox{$\smash{\scriptscriptstyle\frown}$}}{w} = 0,\quad - \overset{\lower0.5em\hbox{$\smash{\scriptscriptstyle\frown}$}}{k}_{hnf} \frac{{\partial \overset{\lower0.5em\hbox{$\smash{\scriptscriptstyle\frown}$}}{T} }}{{\partial \overset{\lower0.5em\hbox{$\smash{\scriptscriptstyle\frown}$}}{z} }} = \overset{\lower0.5em\hbox{$\smash{\scriptscriptstyle\frown}$}}{h}_{{\overset{\lower0.5em\hbox{$\smash{\scriptscriptstyle\frown}$}}{f} }} \left( {\overset{\lower0.5em\hbox{$\smash{\scriptscriptstyle\frown}$}}{T}_{w} - \overset{\lower0.5em\hbox{$\smash{\scriptscriptstyle\frown}$}}{T} } \right),\quad \overset{\lower0.5em\hbox{$\smash{\scriptscriptstyle\frown}$}}{D}_{{\overset{\lower0.5em\hbox{$\smash{\scriptscriptstyle\frown}$}}{A} }} \frac{{\partial \overset{\lower0.5em\hbox{$\smash{\scriptscriptstyle\frown}$}}{A} }}{{\partial \overset{\lower0.5em\hbox{$\smash{\scriptscriptstyle\frown}$}}{z} }} = K_{S} \overset{\lower0.5em\hbox{$\smash{\scriptscriptstyle\frown}$}}{A} ,\quad \overset{\lower0.5em\hbox{$\smash{\scriptscriptstyle\frown}$}}{D}_{{\overset{\lower0.5em\hbox{$\smash{\scriptscriptstyle\frown}$}}{B} }} \frac{{\partial \overset{\lower0.5em\hbox{$\smash{\scriptscriptstyle\frown}$}}{B} }}{{\partial \overset{\lower0.5em\hbox{$\smash{\scriptscriptstyle\frown}$}}{z} }} = - K_{S} \overset{\lower0.5em\hbox{$\smash{\scriptscriptstyle\frown}$}}{A} \,\,at\,z = 0, \hfill \\ \quad \overset{\lower0.5em\hbox{$\smash{\scriptscriptstyle\frown}$}}{u} \to 0,\quad \overset{\lower0.5em\hbox{$\smash{\scriptscriptstyle\frown}$}}{v} \to 0,\quad \overset{\lower0.5em\hbox{$\smash{\scriptscriptstyle\frown}$}}{T} \to \overset{\lower0.5em\hbox{$\smash{\scriptscriptstyle\frown}$}}{T}_{\infty } ,\quad \overset{\lower0.5em\hbox{$\smash{\scriptscriptstyle\frown}$}}{A} \to \overset{\lower0.5em\hbox{$\smash{\scriptscriptstyle\frown}$}}{A}_{0} \quad \overset{\lower0.5em\hbox{$\smash{\scriptscriptstyle\frown}$}}{B} \to 0\,\,as\,\,z \to \infty . \hfill \\ \end{gathered} \right\} $$

The radiative heat flux is deliberated as:

8 $$ \overset{\lower0.5em\hbox{$\smash{\scriptscriptstyle\frown}$}}{q}_{r} = - \frac{{4\sigma^{*} }}{{3k^{*} }}\frac{{\partial \overset{\lower0.5em\hbox{$\smash{\scriptscriptstyle\frown}$}}{T}^{4} }}{{\partial \overset{\lower0.5em\hbox{$\smash{\scriptscriptstyle\frown}$}}{z} }} = - \frac{{16\sigma^{*} \overset{\lower0.5em\hbox{$\smash{\scriptscriptstyle\frown}$}}{T}_{\infty }^{3} }}{{3k^{*} }}\frac{{\partial \overset{\lower0.5em\hbox{$\smash{\scriptscriptstyle\frown}$}}{T} }}{{\partial \overset{\lower0.5em\hbox{$\smash{\scriptscriptstyle\frown}$}}{z} }}. $$

The hybrid nanofluid thermophysical properties are defined as:

9 $$ \left\{ \begin{gathered} \frac{{\mu_{hnf} }}{{\mu_{f} }} = \frac{1}{{\left( {1 - \varphi_{1} - \varphi_{2} } \right)^{2.5} }},\,\,\,\frac{{\rho_{hnf} }}{{\rho_{f} }} = \left( {1 - \varphi_{2} } \right)\left[ {\left( {1 - \varphi_{1} } \right) + \frac{{\varphi_{1} \rho_{1} }}{{\rho_{f} }}} \right] + \frac{{\rho_{2} \varphi_{2} }}{{\rho_{f} }} \hfill \\ \frac{{\left( {\rho C_{p} } \right)_{hnf} }}{{\left( {\rho C_{p} } \right)_{f} }} = \left( {1 - \varphi_{2} } \right)\left[ {\left( {1 - \varphi_{1} } \right) + \frac{{\left( {\rho C_{p} } \right)_{1} \varphi_{1} }}{{\left( {\rho C_{p} } \right)_{f} }}} \right] + \frac{{\left( {\rho C_{p} } \right)_{2} \varphi_{2} }}{{\left( {\rho C_{p} } \right)_{f} }}, \hfill \\ \frac{{\sigma_{hnf} }}{{\sigma_{f} }} = 1 + \frac{{3\left( {\varphi_{1} + \varphi_{2} } \right)\left[ {\varphi_{1} \sigma_{1} + \varphi_{2} \sigma_{2} - \sigma_{f} \left( {\varphi_{1} + \varphi_{2} } \right)} \right]}}{{\varphi_{1} \sigma_{1} + \varphi_{2} \sigma_{2} + 2\sigma_{f} \left( {\varphi_{1} + \varphi_{2} } \right) - \sigma_{f} \left( {\varphi_{1} + \varphi_{2} } \right)\left[ {\varphi_{1} \sigma_{1} + \varphi_{2} \sigma_{2} - \sigma_{f} \left( {\varphi_{1} + \varphi_{2} } \right)} \right]}}, \hfill \\ \frac{{k_{hnf} }}{{k_{{H_{2} O}} }} = 1 + \frac{{3\left( {\varphi_{1} + \varphi_{2} } \right)\left[ {\varphi_{1} k_{1} + \varphi_{2} k_{2} - k_{f} \left( {\varphi_{1} + \varphi_{2} } \right)} \right]}}{{\varphi_{1} k_{1} + \varphi_{2} k_{2} + 2k_{f} \left( {\varphi_{1} + \varphi_{2} } \right) - k_{f} \left( {\varphi_{1} + \varphi_{2} } \right)\left[ {\varphi_{1} k_{1} + \varphi_{2} k_{2} - k_{f} \left( {\varphi_{!} + \varphi_{2} } \right)} \right]}}. \hfill \\ \end{gathered} \right\} $$

The correspondence variables are defined as:

10 $$ \left\{ {\zeta = \sqrt {\frac{{\overset{\lower0.5em\hbox{$\smash{\scriptscriptstyle\frown}$}}{\Omega } }}{{\overset{\lower0.5em\hbox{$\smash{\scriptscriptstyle\frown}$}}{\upsilon }_{f} }}} \overset{\lower0.5em\hbox{$\smash{\scriptscriptstyle\frown}$}}{z} ,\quad \overset{\lower0.5em\hbox{$\smash{\scriptscriptstyle\frown}$}}{u} = \overset{\lower0.5em\hbox{$\smash{\scriptscriptstyle\frown}$}}{r} \overset{\lower0.5em\hbox{$\smash{\scriptscriptstyle\frown}$}}{\Omega } f\left( \zeta \right),\quad \overset{\lower0.5em\hbox{$\smash{\scriptscriptstyle\frown}$}}{v} = \overset{\lower0.5em\hbox{$\smash{\scriptscriptstyle\frown}$}}{r} \overset{\lower0.5em\hbox{$\smash{\scriptscriptstyle\frown}$}}{\Omega } g\left( \zeta \right),\quad \overset{\lower0.5em\hbox{$\smash{\scriptscriptstyle\frown}$}}{w} = \sqrt {\overset{\lower0.5em\hbox{$\smash{\scriptscriptstyle\frown}$}}{\Omega } \overset{\lower0.5em\hbox{$\smash{\scriptscriptstyle\frown}$}}{\upsilon }_{f} } h\left( \zeta \right),\quad \theta \left( \zeta \right) = \frac{{\overset{\lower0.5em\hbox{$\smash{\scriptscriptstyle\frown}$}}{T} - \overset{\lower0.5em\hbox{$\smash{\scriptscriptstyle\frown}$}}{T}_{\infty } }}{{\overset{\lower0.5em\hbox{$\smash{\scriptscriptstyle\frown}$}}{T}_{w} - \overset{\lower0.5em\hbox{$\smash{\scriptscriptstyle\frown}$}}{T}_{\infty } }},\quad \overset{\lower0.5em\hbox{$\smash{\scriptscriptstyle\frown}$}}{A} = \overset{\lower0.5em\hbox{$\smash{\scriptscriptstyle\frown}$}}{A}_{0} I\left( \zeta \right),\quad \overset{\lower0.5em\hbox{$\smash{\scriptscriptstyle\frown}$}}{B} = \overset{\lower0.5em\hbox{$\smash{\scriptscriptstyle\frown}$}}{A}_{0} J\left( \zeta \right)} \right\}. $$

The non-dimensional form of the Eqs. (1–6) are obtained by applying the above-mentioned similarity transformation in Eq. (10):

11 $$ h^{\prime } + 2f = 0, $$

12 $$ \frac{{\mu_{hnf} /\mu_{f} }}{{\rho_{hnf} /\rho_{f} }}f^{\prime \prime } - (f^{\prime 2} - g^{2} + hf^{\prime } ) - \frac{{\sigma_{hnf} /\sigma_{f} }}{{\rho_{hnf} /\rho_{f} }}Mf + \frac{{\left( {\rho \beta } \right)_{hnf} }}{{\left( {\rho \beta } \right)_{f} }}\lambda \theta = 0, $$

13 $$ \frac{{\mu_{hnf} /\mu_{f} }}{{\rho_{hnf} /\rho_{f} }}g^{\prime \prime } - \left( {2fg + hg^{\prime } } \right) - \frac{{\sigma_{hnf} /\sigma_{f} }}{{\rho_{hnf} /\rho_{f} }}Mg = 0, $$

14 $$ \frac{{\left( {\rho C_{p} } \right)_{f} }}{{\left( {\rho C_{p} } \right)_{hnf} }}\left( {\frac{{k_{hnf} }}{{k_{f} }} + Rd} \right)\theta^{\prime \prime } - \Pr h\theta^{\prime } = 0, $$

15 $$ \frac{1}{Sc}I^{\prime \prime } - K_{1} IJ^{2} - hI^{\prime } = 0, $$

16 $$ \frac{\delta }{Sc}J^{\prime \prime } + K_{1} IJ^{2} - hJ^{\prime } = 0, $$

With boundary conditions:

17 $$ \left\{ \begin{gathered} f\left( 0 \right),\quad g\left( 0 \right) = 1,\quad \frac{{\overset{\lower0.5em\hbox{$\smash{\scriptscriptstyle\frown}$}}{k}_{hnf} }}{{\overset{\lower0.5em\hbox{$\smash{\scriptscriptstyle\frown}$}}{k}_{f} }}\theta^{\prime } \left( 0 \right) = Bi_{T} \left( {\theta \left( 0 \right) - 1} \right),\quad I^{\prime } \left( 0 \right) = K_{2} I\left( 0 \right),\quad J^{\prime } \left( 0 \right) = - K_{2} J\left( 0 \right), \hfill \\ f\left( \infty \right) \to 0,\quad g\left( \infty \right) \to 0,\quad \theta \left( \infty \right) \to 0,\quad I\left( \infty \right) \to 1,\quad J\left( \infty \right) \to 0. \hfill \\ \end{gathered} \right\} $$

Further, it is assumed that the ratio of the diffusion coefficient $D_{A}$ and $D_{B}$ are to be comparable, therefore ratio of diffusion coefficient is assumed to be 1, so $\delta = 1$ and $I\left( \zeta \right) + J\left( \zeta \right) = 1$.

Therefore, Eqs. (15) and (16) are reduced as:

18 $$ \frac{1}{Sc}I^{\prime \prime } - hI^{\prime } - K_{1} I\left( {1 + I^{2} - 2I} \right) = 0, $$

with boundary conditions:

19 $$ I^{\prime } \left( 0 \right) = K_{2} I\left( 0 \right),\quad I\left( \infty \right) \to 1. $$

After the simplification, some significant dimensionless parameters are discussed here. The magnetic field is denoted by $$M\left( { = \frac{{\overset{\lower0.5em\hbox{$\smash{\scriptscriptstyle\frown}$}}{\sigma }_{f} B_{0}^{2} }}{{\overset{\lower0.5em\hbox{$\smash{\scriptscriptstyle\frown}$}}{\rho }_{f} \overset{\lower0.5em\hbox{$\smash{\scriptscriptstyle\frown}$}}{\Omega } }}} \right)$$, $$\lambda \left( { = \frac{{\overset{\lower0.5em\hbox{$\smash{\scriptscriptstyle\frown}$}}{g} \overset{\lower0.5em\hbox{$\smash{\scriptscriptstyle\frown}$}}{\beta }_{f} \left( {\overset{\lower0.5em\hbox{$\smash{\scriptscriptstyle\frown}$}}{T}_{w} - \overset{\lower0.5em\hbox{$\smash{\scriptscriptstyle\frown}$}}{T}_{\infty } } \right)}}{{\overset{\lower0.5em\hbox{$\smash{\scriptscriptstyle\frown}$}}{r} \overset{\lower0.5em\hbox{$\smash{\scriptscriptstyle\frown}$}}{\Omega }^{2} }}} \right)$$ is the mixed convention parameter, Prandtl number is represented by $$\Pr \left( { = \frac{{\left( {\overset{\lower0.5em\hbox{$\smash{\scriptscriptstyle\frown}$}}{\rho } \overset{\lower0.5em\hbox{$\smash{\scriptscriptstyle\frown}$}}{C}_{{\overset{\lower0.5em\hbox{$\smash{\scriptscriptstyle\frown}$}}{p} }} } \right)_{f} \overset{\lower0.5em\hbox{$\smash{\scriptscriptstyle\frown}$}}{\upsilon }_{f} }}{{\overset{\lower0.5em\hbox{$\smash{\scriptscriptstyle\frown}$}}{k}_{f} }}} \right)$$, $$Rd\left( { = \frac{{16\sigma^{*} \overset{\lower0.5em\hbox{$\smash{\scriptscriptstyle\frown}$}}{T}_{\infty }^{3} }}{{3k^{*} \overset{\lower0.5em\hbox{$\smash{\scriptscriptstyle\frown}$}}{k}_{f} }}} \right)$$ is the radiation parameter, $$Sc\left( { = \frac{{\overset{\lower0.5em\hbox{$\smash{\scriptscriptstyle\frown}$}}{\upsilon }_{f} }}{{\overset{\lower0.5em\hbox{$\smash{\scriptscriptstyle\frown}$}}{D}_{{\overset{\lower0.5em\hbox{$\smash{\scriptscriptstyle\frown}$}}{A} }} }}} \right)$$ is the Schmidt number, homogeneous chemical reaction parameter is symbolized by $$K_{1} \left( { = \frac{{\overset{\lower0.5em\hbox{$\smash{\scriptscriptstyle\frown}$}}{K}_{C} \overset{\lower0.5em\hbox{$\smash{\scriptscriptstyle\frown}$}}{A}_{0}^{2} }}{{\overset{\lower0.5em\hbox{$\smash{\scriptscriptstyle\frown}$}}{\Omega } }}} \right)$$, heterogeneous chemical reaction is denoted by $$K_{2} \left( { = \frac{{\overset{\lower0.5em\hbox{$\smash{\scriptscriptstyle\frown}$}}{K}_{S} }}{{\overset{\lower0.5em\hbox{$\smash{\scriptscriptstyle\frown}$}}{D}_{{\overset{\lower0.5em\hbox{$\smash{\scriptscriptstyle\frown}$}}{A} }} }}\sqrt {\frac{{\overset{\lower0.5em\hbox{$\smash{\scriptscriptstyle\frown}$}}{\upsilon }_{f} }}{{\overset{\lower0.5em\hbox{$\smash{\scriptscriptstyle\frown}$}}{\Omega } }}} } \right)$$, $$Bi_{T} \left( { = \frac{{\overset{\lower0.5em\hbox{$\smash{\scriptscriptstyle\frown}$}}{h}_{f} }}{{\overset{\lower0.5em\hbox{$\smash{\scriptscriptstyle\frown}$}}{k}_{f} }}\sqrt {\frac{{\overset{\lower0.5em\hbox{$\smash{\scriptscriptstyle\frown}$}}{\upsilon }_{f} }}{{\overset{\lower0.5em\hbox{$\smash{\scriptscriptstyle\frown}$}}{\Omega } }}} } \right)$$ is the thermal Biot number, and the ratio of diffusion coefficient is expressed by $$\delta \left( { = \frac{{\overset{\lower0.5em\hbox{$\smash{\scriptscriptstyle\frown}$}}{D}_{{\overset{\lower0.5em\hbox{$\smash{\scriptscriptstyle\frown}$}}{B} }} }}{{\overset{\lower0.5em\hbox{$\smash{\scriptscriptstyle\frown}$}}{D}_{{\overset{\lower0.5em\hbox{$\smash{\scriptscriptstyle\frown}$}}{A} }} }}} \right)$$.

The $C_{{f\tilde{r}}}$ and $Nu_{r}$ are defined as^46,47^:

20 $$ \left\{ \begin{gathered} C_{fr} = \frac{1}{{\overset{\lower0.5em\hbox{$\smash{\scriptscriptstyle\frown}$}}{\rho }_{f} \overset{\lower0.5em\hbox{$\smash{\scriptscriptstyle\frown}$}}{u}_{w}^{2} }}\sqrt {\overset{\lower0.5em\hbox{$\smash{\scriptscriptstyle\frown}$}}{\tau }_{{\overset{\lower0.5em\hbox{$\smash{\scriptscriptstyle\frown}$}}{r} }}^{2} + \overset{\lower0.5em\hbox{$\smash{\scriptscriptstyle\frown}$}}{\tau }_{{^{{\overset{\lower0.5em\hbox{$\smash{\scriptscriptstyle\frown}$}}{\vartheta } }} }}^{2} } , \hfill \\ Nu_{r} = \frac{{\overset{\lower0.5em\hbox{$\smash{\scriptscriptstyle\frown}$}}{r} \overset{\lower0.5em\hbox{$\smash{\scriptscriptstyle\frown}$}}{q}_{{\overset{\lower0.5em\hbox{$\smash{\scriptscriptstyle\frown}$}}{w} }} }}{{\overset{\lower0.5em\hbox{$\smash{\scriptscriptstyle\frown}$}}{k}_{f} \left( {\overset{\lower0.5em\hbox{$\smash{\scriptscriptstyle\frown}$}}{T}_{w} - \overset{\lower0.5em\hbox{$\smash{\scriptscriptstyle\frown}$}}{T}_{\infty } } \right)}},\, \hfill \\ \end{gathered} \right\} $$

where $$\overset{\lower0.5em\hbox{$\smash{\scriptscriptstyle\frown}$}}{\tau }_{{\overset{\lower0.5em\hbox{$\smash{\scriptscriptstyle\frown}$}}{r} }} = \overset{\lower0.5em\hbox{$\smash{\scriptscriptstyle\frown}$}}{\mu }_{hnf} \left. {\left( {\frac{{\partial \overset{\lower0.5em\hbox{$\smash{\scriptscriptstyle\frown}$}}{u} }}{{\partial \overset{\lower0.5em\hbox{$\smash{\scriptscriptstyle\frown}$}}{z} }} + \frac{{\partial \overset{\lower0.5em\hbox{$\smash{\scriptscriptstyle\frown}$}}{w} }}{{\partial \overset{\lower0.5em\hbox{$\smash{\scriptscriptstyle\frown}$}}{r} }}} \right)} \right|_{{\overset{\lower0.5em\hbox{$\smash{\scriptscriptstyle\frown}$}}{z} = 0}}$$, $$\overset{\lower0.5em\hbox{$\smash{\scriptscriptstyle\frown}$}}{\tau }_{{\overset{\lower0.5em\hbox{$\smash{\scriptscriptstyle\frown}$}}{\varphi } }} = \overset{\lower0.5em\hbox{$\smash{\scriptscriptstyle\frown}$}}{\mu }_{hnf} \left. {\left( {\frac{{\partial \overset{\lower0.5em\hbox{$\smash{\scriptscriptstyle\frown}$}}{v} }}{{\partial \overset{\lower0.5em\hbox{$\smash{\scriptscriptstyle\frown}$}}{z} }} + \frac{1}{{\overset{\lower0.5em\hbox{$\smash{\scriptscriptstyle\frown}$}}{r} }}\frac{{\partial \overset{\lower0.5em\hbox{$\smash{\scriptscriptstyle\frown}$}}{w} }}{\partial r}} \right)} \right|_{{\overset{\lower0.5em\hbox{$\smash{\scriptscriptstyle\frown}$}}{z} = 0}}$$ and $$\overset{\lower0.5em\hbox{$\smash{\scriptscriptstyle\frown}$}}{q}_{w} = - \overset{\lower0.5em\hbox{$\smash{\scriptscriptstyle\frown}$}}{k}_{hnf} \left. {\frac{{\partial \overset{\lower0.5em\hbox{$\smash{\scriptscriptstyle\frown}$}}{T} }}{{\partial \overset{\lower0.5em\hbox{$\smash{\scriptscriptstyle\frown}$}}{z} }}} \right|_{{\overset{\lower0.5em\hbox{$\smash{\scriptscriptstyle\frown}$}}{z} = 0}} + \left. {q_{r} } \right|_{{\overset{\lower0.5em\hbox{$\smash{\scriptscriptstyle\frown}$}}{z} = 0}}$$.

In dimensionless form, $C_{{f\tilde{r}}}$ and $Nu_{r}$ are:

21 $$ \left\{ \begin{gathered} {\text{Re}}_{x}^{\frac{1}{2}} C_{fr} = \frac{1}{{\left( {1 - \phi_{1} } \right)^{2.5} \left( {1 - \phi_{2} } \right)^{2.5} }}\left( {f^{\prime \prime 2} \left( 0 \right) + g^{\prime 2} \left( 0 \right)} \right)^{1/2} , \hfill \\ {\text{Re}}_{x}^{{ - \frac{1}{2}}} Nu_{r} = - \left( {\frac{{k_{hnf} }}{{k_{f} }} + Rd} \right)\theta^{\prime } , \hfill \\ \end{gathered} \right\} $$

where $${\text{Re}}_{x} \left( { = \frac{{\overset{\lower0.5em\hbox{$\smash{\scriptscriptstyle\frown}$}}{\Omega } \overset{\lower0.5em\hbox{$\smash{\scriptscriptstyle\frown}$}}{r}^{2} }}{{\overset{\lower0.5em\hbox{$\smash{\scriptscriptstyle\frown}$}}{\upsilon }_{f} }}} \right)$$ is the local Reynolds number.

## HAM solution

The initial guesses are given as:

22 $$ f_{0} \left( \eta \right) = 0,\,\,\,\,g_{0} \left( \eta \right) = e^{{\left( { - \eta } \right)}} ,\,\,\,\,\theta_{0} \left( \eta \right) = \frac{{Bi_{T} }}{{\left( {1 + Bi_{T} } \right)\overset{\lower0.5em\hbox{$\smash{\scriptscriptstyle\frown}$}}{k}_{hnf} /\overset{\lower0.5em\hbox{$\smash{\scriptscriptstyle\frown}$}}{k}_{f} }}e^{{\left( { - \eta } \right)}} ,\,\,\,\,I_{0} \left( \eta \right) = 1 - \frac{1}{2}e^{{\left( { - K_{2} \eta } \right)}} , $$

The linear operators are given as:

23 $$ L_{f} = f^{\prime \prime } - f,\quad L_{g} = g^{\prime \prime } - g,\quad L_{\theta } = \theta^{\prime \prime } - \theta ,\quad L_{I} = I^{\prime \prime } - I, $$

with

24 $$ L_{f} \left[ {\alpha_{1} e^{ - \eta } + \alpha_{2} e^{\eta } } \right] = 0,\quad L_{g} \left[ {\alpha_{3} e^{ - \eta } + \alpha_{4} e^{\eta } } \right] = 0,\quad L_{\theta } \left[ {\alpha_{5} e^{ - \eta } + \alpha_{6} e^{\eta } } \right] = 0,\quad L_{I} \left[ {\alpha_{7} e^{ - \eta } + \alpha_{8} e^{\eta } } \right] = 0, $$

where $\alpha_{n} \left( {n = 1 - 8} \right)$ are called the arbitrary constants.

### HAM convergence

**Figure 1:**
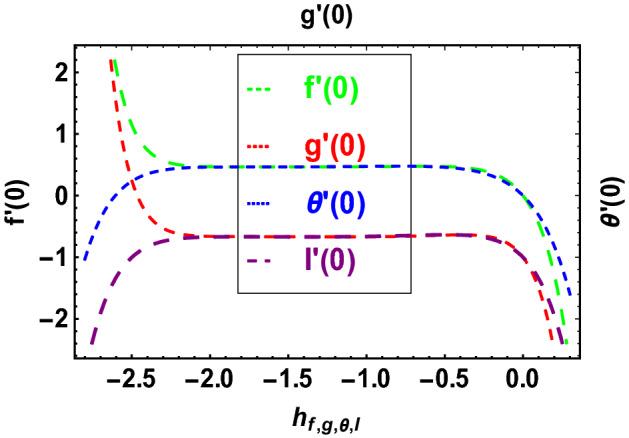
$\hbar$**-**curves for $f^{\prime}\left( 0 \right)$, $g^{\prime}\left( 0 \right)$, $\theta^{\prime}\left( 0 \right)$ and $I^{\prime}\left( 0 \right)$.

Convergence of the modeled equations is ensured through the use of HAM. The parameter $\hbar$ is crucial to the model system of equations' convergence. Figure 1 depicts the convergence regions for the velocities, thermal, and concentration profiles at the 18th order of approximation. The convergence regions of velocities, temperature and concentration profiles respectively are $- 2.1 \le \hbar_{f} \le 0.2$, $- 2.1 \le \hbar_{g} \le 0.2$, $- 2.2 \le \hbar_{\theta } \le 0.2$ and $- 2.2 \le \hbar_{I} \le 0.2$.

## Results and discussion

**Figure 2:**
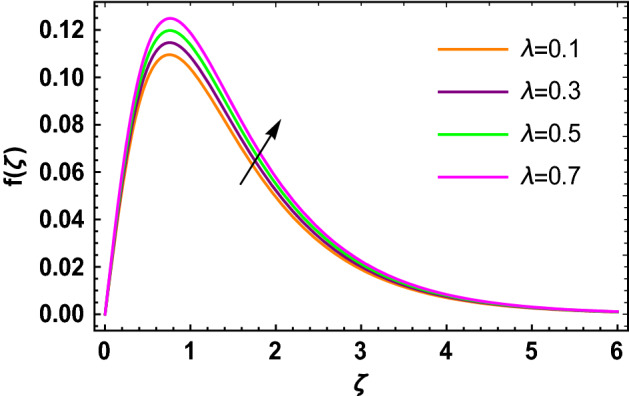
$$f\left( \zeta \right)$$ versus $$\lambda$$.

**Figure 3:**
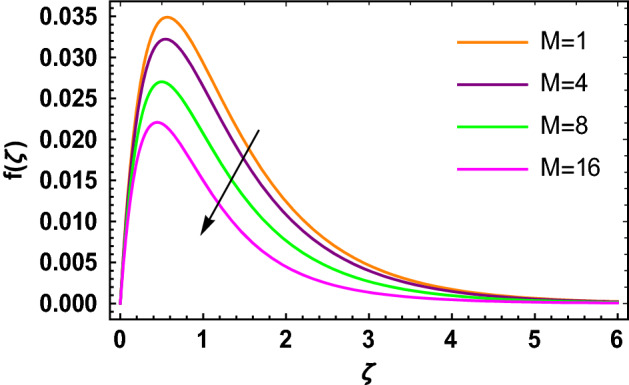
$$f\left( \zeta \right)$$ versus $$M$$.

**Figure 4:**
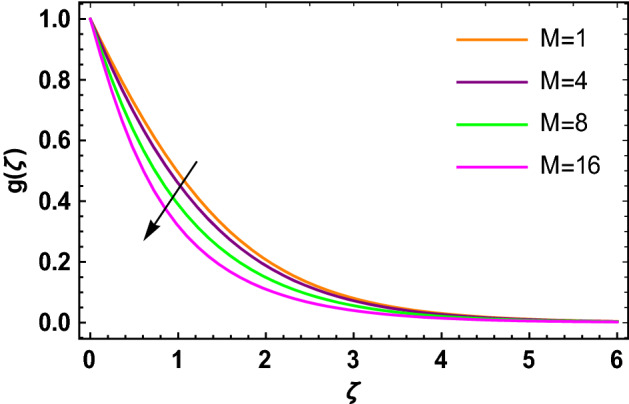
$$g\left( \zeta \right)$$ versus $$M$$.

**Figure 5:**
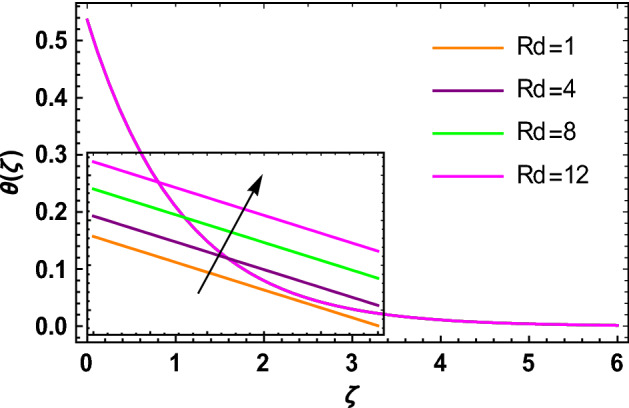
$$\theta \left( \zeta \right)$$ versus $$Rd$$.

**Figure 6:**
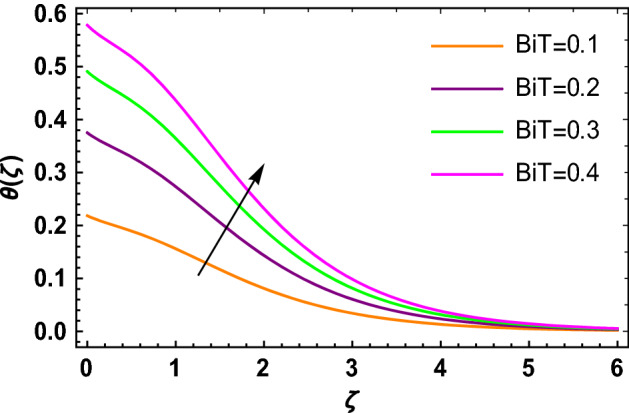
$$\theta \left( \zeta \right)$$ versus $$Bi_{T}$$.

**Figure 7:**
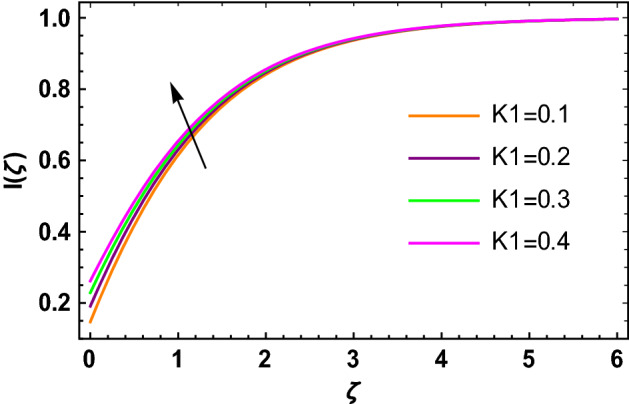
$$I\left( \zeta \right)$$ versus $$K_{1}$$.

**Figure 8:**
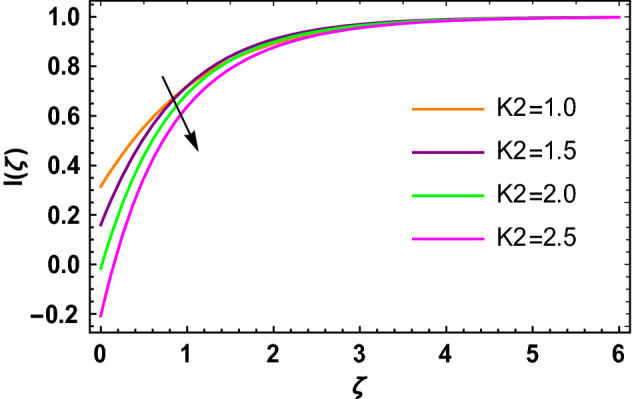
$$I\left( \zeta \right)$$ versus $$K_{2}$$.

**Figure 9:**
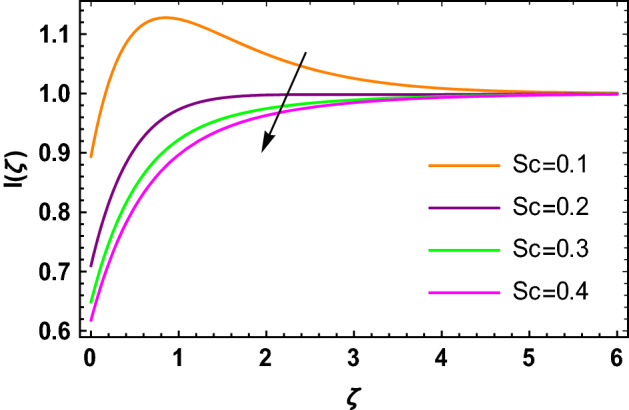
$$I\left( \zeta \right)$$ versus $$Sc$$.

This portion determined the physical aspect of several flow parameters on velocities, temperature, skin friction coefficient and Nusselt number of the hybrid nanofluid. Table 1 shows the thermophysical characteristic of the water, ethylene glycol and graphene nanoparticles. Table 2 shows the comparison of the present investigation with previous studies. Here, we have found a great agreement with previous studies. Table 3 explains the fluctuation of the skin friction coefficient $C_{fr}$ of the hybrid nanofluid via magnetic field parameter $M$, mixed convection parameter $\lambda$ and nanoparticles volume fraction $\phi_{1} + \phi_{2}$. It has noted from Table 2 that $C_{fr}$ is higher when $M$, $\lambda$ and $\phi_{1} + \phi_{2}$ are rises. In Table 4, the role of the radiation parameter $Rd$, thermal Biot number $Bi_{T}$ and nanoparticles volume fraction $\phi_{1} + \phi_{2}$ on the Nusselt number $Nu_{r}$ is deliberated. In this investigation, it is revealed that with the increase of $Rd$, $Bi_{T}$ and $\phi_{1} + \phi_{2}$, the Nusselt number $Nu_{r}$ is increases. Figure 2 displays the influence of mixed convection parameter $\lambda$ on the primary velocity of the hybrid nanofluid flow. This figure determines that the nanofluid primary velocity increases with the increase in $\lambda$. This is due to the fact that when the buoyancy on free convection gets considerable, a mixed convection occurs. Therefore, the buoyancy will grow if the value of $\lambda$ is enlarged. The flow velocity increased as the buoyancy force does as well. The role of the primary velocity of the hybrid nanofluid flow versus magnetic parameter $M$ is discussed in Fig. 3. It is interpreted that the enhancement in $M$ led to a decline in the primary velocity of the hybrid nanofluid. This effect is due to the reason that as we increase $M$, so does the Lorentz force. Lorentz force is actually retarding force acting against the velocity profile. So, the increasing $M$ increases the opposing force (as shown in Table 3) to the hybrid nanofluid flow which results a reduction in the velocity profile of the hybrid nanofluid flow. Figure 4 explores the influence of the magnetic field parameter on the secondary velocity profile of the hybrid nanofluid. The decayed in secondary velocity of the hybrid nanofluid is noticed for higher estimates of the magnetic parameter. Further, it is due to the fact that the rising values of $M$ produce a Lorentz force. So, the production of the Lorentz force slows down the liquid particles motion that’s why the velocity profile is diminished. Figure 5 demonstrates the fluctuation in temperature profile of the hybrid nanoliquid due to the rising of the radiation parameter $Rd$. It is eminent that an augmentation in radiation parameter upsurges the temperature of the hybrid nanoliquid. Since the radiation parameter and the mean absorption coefficient are inversely proportional, more heat is transmitted to the working liquid via the radiation phenomenon. The temperature increases as $k^{*}$ decreases with increasing $Rd$ values. Both the rate of thermal convection into the fluid and the deviation of radiative heat flow increase as a result. The growth of thermal boundary layers derives from the improved thermal heat transmission. The effect is a thickening of the thermal layer. The effect of the thermal Biot number on the temperature of the hybrid nanoliquid is shown in Fig. 6. Figure 6 explains that as the values of the thermal Biot number increase, the hybrid nanofluid temperature also increases. Further, on the rotating disk, the thermal Biot number is connected with the convective condition. Also, the thermal boundary layer thickness is enhancing due to the implementation of the convective condition. So, near the surface of the disk, the hybrid nanoliquid temperature increases when the thermal Biot number rises. The consequence of the homogeneous chemical reaction parameter on the hybrid nanoliquid concentration profile is discussed in Fig. 7. In this investigation, it is observed that the concentration of the hybrid nanofluid is enhanced with the rising of the homogeneous chemical reaction parameter. For different values of the heterogeneous chemical reaction parameter, the behavior of hybrid nanoliquid concentration is deliberated in Fig. 8. It is evident that the concentration of the hybrid nanofluid declines due to the increment in the heterogeneous chemical reaction parameter. In Fig. 9, the role of the Schmidt number $Sc$ on the concentration of the hybrid nanoliquid is identified. In this figure, it is recognized that the hybrid nanofluid concentration is lesser for higher values of $Sc$. Generally, $Sc$ is defined as the ratio between the momentum diffusivity and mass diffusivity. So, with the increase of $Sc$, the mass diffusivity of the fluid is small. Therefore the concentration of the hybrid nanofluid is lesser due to the increase in $Sc$.

**Table 1:** Thermophysical characteristic of the water, ethylene glycol and graphene nanoparticles^41–45^.

Thermophysical properties	$$\overset{\lower0.5em\hbox{$\smash{\scriptscriptstyle\frown}$}}{\rho }$$	$$\overset{\lower0.5em\hbox{$\smash{\scriptscriptstyle\frown}$}}{C}_{{\overset{\lower0.5em\hbox{$\smash{\scriptscriptstyle\frown}$}}{p} }}$$	$$\overset{\lower0.5em\hbox{$\smash{\scriptscriptstyle\frown}$}}{k}$$	$$\overset{\lower0.5em\hbox{$\smash{\scriptscriptstyle\frown}$}}{\sigma }$$	$$\beta$$	$$Pr$$
50%W + 50%EG	1056	3288	0.425	0.00509	0.00341	29.86
Graphene	2250	2100	2500	1 × 10^7^	2.84 × 10^−4^	–
Silver	10,500	235	429	63 × 10^−6^	1.89 × 10^−5^	–

**Table 2:** Comparison of $- \theta^{\prime}\left( 0 \right)$ with reported results when all other parameters are zero.

$\phi_{1} + \phi_{2}$	$\Pr$	Bachok et al.^48^	Maleque and Sattar^49^	Kelson and Desseaux^50^	Present results
0.0	0.71	0.3259	0.325769	0.325856	0.3258

**Table 3:** Chang in $C_{fr}$ due to $M$, $\lambda$ and $\phi_{1} + \phi_{2}$.

$M$	$\lambda$	$\phi_{1} + \phi_{2}$	$C_{fr}$
0.1			1.670699
0.3			1.671767
0.5			1.672835
0.7			1.673903
	0.2		1.670767
	0.4		1.670905
	0.6		1.671042
	0.8		1.671180
		0.01	1.224952
		0.02	1.281557
		0.03	1.343170
		0.04	1.409918

**Table 4:** Chang in $Nu_{r}$ due to $Rd$, $Bi_{T}$ and $\phi_{1} + \phi_{2}$.

$Rd$	$Bi_{T}$	$\phi_{1} + \phi_{2}$	$Nu_{r}$
1.0			0.152344
1.5			0.152346
2.0			0.152348
2.5			0.152349
	1.0		1.720965
	2.0		2.183312
	3.0		3.249004
	4.0		3.893915
		0.01	0.109616
		0.02	0.118770
		0.03	0.128756
		0.04	0.139692

## Conclusion

In this investigation, MHD flow of hybrid nanofluid containing graphene and silver nanoparticles with water and ethylene glycol (50:50) over a rotating disk is analyzed. To explore the idea of heat transport, simulation for thermal radiation under convective condition is performed. Furthermore, the homogeneous and heterogeneous chemical reactions are discussed in the present flow phenomenon. On the basis of HAM method, solution of the modeled problem is performed. It is detected that hybrid nanofluid skin friction coefficient is enhanced due to magnetic parameter, mixed convection parameter and nanoparticles volume fraction. Intensification in Nusselt number is noticed for higher radiation parameter, thermal Biot number and nanoparticles volume fraction. Escalation in mixed convection parameter increases the velocity profile, while reduces with the increasing magnetic parameter. With the rising of radiation parameter and thermal Biot number, hybrid nanofluid temperature gets higher. Hybrid nanofluid concentration is greater for homogeneous chemical reaction parameter. Also, it is noted that the higher heterogeneous chemical reaction parameter and Schmidt number lowers the hybrid nanofluid concentration.

## Data Availability

All data used in this manuscript have been presented within the article.

## Abbreviations

(*u*, *v*, *w*): Velocity components
$$\left( {\overset{\lower0.5em\hbox{$\smash{\scriptscriptstyle\frown}$}}{r} ,\overset{\lower0.5em\hbox{$\smash{\scriptscriptstyle\frown}$}}{\vartheta } ,\overset{\lower0.5em\hbox{$\smash{\scriptscriptstyle\frown}$}}{z} } \right)$$: Cylindrical coordinate system
$B_{0}$: Magnetic field strength
$$\overset{\lower0.5em\hbox{$\smash{\scriptscriptstyle\frown}$}}{T}$$: Temperature
EG: Ethylene glycol
$$g$$: Gravity
$k^{*}$: Mean absorption coefficient
$$ \overset{\lower0.5em\hbox{$\smash{\scriptscriptstyle\frown}$}}{k} $$: Thermal conductivity
$$\overset{\lower0.5em\hbox{$\smash{\scriptscriptstyle\frown}$}}{q}_{r}$$: Radiative heat flux
$$\overset{\lower0.5em\hbox{$\smash{\scriptscriptstyle\frown}$}}{K}_{C}$$ and $$K_{S}$$: Chemical reaction rate
$$\overset{\lower0.5em\hbox{$\smash{\scriptscriptstyle\frown}$}}{D}_{{\overset{\lower0.5em\hbox{$\smash{\scriptscriptstyle\frown}$}}{A} }}$$ and $$\overset{\lower0.5em\hbox{$\smash{\scriptscriptstyle\frown}$}}{D}_{{\overset{\lower0.5em\hbox{$\smash{\scriptscriptstyle\frown}$}}{B} }}$$: Diffusion coefficient
$$ \overset{\lower0.5em\hbox{$\smash{\scriptscriptstyle\frown}$}}{A} \;{\text{and}}\;\overset{\lower0.5em\hbox{$\smash{\scriptscriptstyle\frown}$}}{B} $$: Chemical species
$f\left( \zeta \right)$, $g\left( \zeta \right)$ and $h\left( \zeta \right)$: Dimensionless velocities
$I$ and $J$: Homogeneous and heterogeneous concentration
$Bi_{T}$: Thermal Biot number
$K_{1}$: Homogeneous chemical reaction parameter
$K_{2}$: Heterogeneous chemical reaction parameter
$M$: Magnetic field parameter
$$\Pr$$: Prandtl number
$$Rd$$: Radiation parameter
$$Sc$$: Schmidt number
$C_{fr}$: Skin friction coefficient
$$Nu_{r}$$: Nusselt number

## Greek letter

$$\theta$$: Temperature
$$\overset{\lower0.5em\hbox{$\smash{\scriptscriptstyle\frown}$}}{\nu }$$: Kinematic viscosity
$$\overset{\lower0.5em\hbox{$\smash{\scriptscriptstyle\frown}$}}{\sigma }$$: Electrical conductivity
$$\overset{\lower0.5em\hbox{$\smash{\scriptscriptstyle\frown}$}}{\rho }$$: Density
$$\overset{\lower0.5em\hbox{$\smash{\scriptscriptstyle\frown}$}}{C}_{{\overset{\lower0.5em\hbox{$\smash{\scriptscriptstyle\frown}$}}{p} }}$$: Specific heat
$\sigma ^{*}$: Stefan-Boltzmann constant
$$\overset{\lower0.5em\hbox{$\smash{\scriptscriptstyle\frown}$}}{\mu }$$: Dynamic viscosity
$\lambda$: Dimensionless mixed convection parameter
$$\overset{\lower0.5em\hbox{$\smash{\scriptscriptstyle\frown}$}}{\beta }$$: Non-dimensional mixed convection parameter
$\delta$: Ratio of diffusion coefficient
$$\overset{\lower0.5em\hbox{$\smash{\scriptscriptstyle\frown}$}}{\Omega }$$: Angular velocity

## Subscript

$$f$$: Fluid
$$hnf$$: Hybrid nanofluid
$w$: At the surface
$$\infty$$: Free stream
